# *Pneumocystis carinii* versus *Pneumocystis jiroveci*: Another Misnomer (Response to Stringer et al.)

**DOI:** 10.3201/eid0902.020602

**Published:** 2003-02

**Authors:** Walter T. Hughes

**Affiliations:** * St. Jude Children’s Research Hospital, Memphis, Tennessee, USA

**Keywords:** Pneumocystis carinii, Pneumocystis jiroveci, Pneumocystis carinii pneumonitis, pneumonia, opportunistic organisms, nomenclature, taxonomy, letter

**To the Editor:** The proposal by Stringer et al. to change the name of *Pneumocystis carinii* found in humans to *Pneumocystis jirovec* requires critical consideration (*1*). First, their rationale for the choice of Jírovec is not compelling. Principle III of the International Code of Botanical Nomenclature (ICBN) states: “the nomenclature of a taxonomic group is based upon priority of publication” (*2*). Jírovec’s publication in 1952 was not the first to report *P. carinii* infection in human lungs. In 1942, two Dutch investigators, van der Meer and Brug, described *P. carinii* as the infecting organism in a 3-month-old infant with congenital heart disease and in 2 of 104 autopsy cases (a 4-month-old infant and a 21-year-old adult) (*3*). Their description, photomicrographs, and drawings of *P. carinii* are unequivocal. They also described the typical “honeycomb” patterns in alveoli. In 1951, Dr. Josef Vanek at Karls-Universität in Praha, Czechoslovakia, reported his study of lung sections from 16 children with interstitial pneumonia and demonstrated that the disease was caused by *P. carinii* (*4*). Vanek notes in his report, “In man the parasite was for the first time established as a cause of pneumonia in a child by G. Meer and S. L. Brug (1942).” In 1952, Jírovec reported *P. carinii* as the cause of interstitial plasmacellular pneumonia in neonates (*5*). A year later, in a coauthored publication, Vanek, Jírovec, and J. Lukes acknowledged and referenced the earlier reports of van der Meer and Brug and Vanek (*6*). If principle III is to be followed, as well as fairness to the investigators, both van der Meer and Brug and Vanek hold priority over Jírovec, assuming the designation of the species name should be based on the name of the first person to discover *P. carinii* in humans.

The nomenclature of *P. carinii* has actually been fraught with errors from the beginning. In the earliest publications, Carlos Chagas and Antonio Carini mistook the organism for stages in the life cycle of trypanosomes. Chagas placed it in a new genus, *Schizotrypanum* (*7*,*8*). In 1912, Delanoë and Delanoë at the Pasteur Institute in Paris published the first description of the organism as a new entity unrelated to trypanosomes (*9*). They proposed the name *“Pneumocystis carinii”* as a tribute to Carini. The Delanoë paper has remained unchallenged as the original description of *P. carinii*. Both Chagas and Carini later acknowledged their errors and the validity of the Delanoës’ conclusion. By current ICBN principles, *P. carinii* is acceptable nomenclature because the authors of the first publication proposed the name of Carini, rather than their own.

In addition, changing the name to *P. jiroveci* will create confusion in clinical medicine where the name *P. carinii* has served physicians and microbiologists well for over half a century. I was moved to write this letter because of a call from a knowledgeable oncologist asking for information on “the new strain of *P. carinii* that has just been reported from the Centers for Disease Control and Prevention,” referring to the report by Stringer et al (*1*).

AIDS patients are well informed about *P. carinii* pneumonia and avidly monitor medical news about their disease. Without doubt, the name change will cause confusion and undue anxiety among the many thousands of HIV-infected patients who attend clinics. Health-care workers will have an added burden of explaining why the name was changed, but the organism and infection are unchanged. Also, versions of the pronunciation of *jiroveci* (yee row vet zee) by American patients, physicians, and health-care workers will be interesting to hear.

The tone of the article by Stringer et al. implies that the change of *P. carinii* to *P. jiroveci* is final, which is not the case. The nomenclature of fungi is governed by ICBN under the auspices of the International Botanical Congress and is not based solely on molecular genetics. Neither *P. carinii* nor *P. jiroveci* have been submitted for ICBN scrutiny. In another paper, Stringer et al. outline the mechanics for submission, but indicate that no application has been submitted for their proposal (*10*). In fact, *P. carinii* has not been acknowledged as a fungus by ICBN or any other authoritative taxonomic system. Only when nomenclature is registered in ICBN, can a name be referred to as “formally accepted.” In the meantime, the workable terminology proposed earlier by Stringer et al. in 1994 (*11*) will suffice for clinical use.
