# Supplementary material for: Pneumocystis carinii versus Pneumocystis jiroveci: Another Misnomer (Response to Stringer et al.)
Source: Emerg Infect Dis. 2003 Feb;9(2):276–7. doi: 10.3201/eid0902.020602 (PMC2901955; doi:10.3201/eid0902.020602)
Supplement: Related texts

# Letter

**DOI:** 10.3201/eid0902.020711

**Published:** 2003-02

**Authors:** James R. Stringer, Charles Ben Beard, Robert F. Miller, Melanie T. Cushion

**Affiliations:** * University of Cincinnati, Cincinnati, Ohio, USA; † Centers for Disease Control and Prevention, Atlanta, Georgia, USA; ‡ University College London, London, UK

## In Response

We appreciate Dr. Hughes’ letter of concern regarding our article endorsing the name *Pneumocystis jiroveci* (*1*). When working with well-known disease agents and syndromes, these types of changes are more difficult to adopt because of the effect they have on daily communication, patient care, record keeping, and other important routines of health-care providers. However, in this case, new information and understanding dictate that a change be made.

For some time, scientists have known that humans are infected by a particular species of *Pneumocystis* and that this species does not infect other host species. In recognition of these facts, Frenkel named the human pathogen *Pneumocystis jiroveci,* using the procedure prescribed by the International Code of Botanical Nomenclature (ICBN) (*2*). Although Dr. Hughes raised a number of issues, none justifies rejecting the new, valid name.

Dr. Hughes suggested that the name *P. jiroveci* is incorrect on the basis of principal III of ICBN, which holds that “the nomenclature of a taxonomic group is based upon priority of publication.” He indicated that Jírovec was not the first investigator to report *Pneumocystis* in humans. Although this situation may be the case, principal III has not been violated because “priority of publication” refers to the time when a name is validly published, not to the time when an organism is first described. The name *P. jiroveci* was validly published in 1999, and this name therefore has priority. To be valid, all of the following steps must be completed: a name must be published in a scientific journal, the name must be a binary Latin name, the organism must be described in Latin, the rank of the organism must be indicated, and the new species must be called by the term “typus or holotypus,”and the specimen or microscope slides must be placed in a public holding (details are available from: URL: http://www.bgbm.fu-berlin.de/iapt/nomenclature/code/SaintLouis/0000St.Luistitle.htm). Dr. Frenkel was the first to fulfill these requirements in his 1999 publication (*2*).

The 1912 publication by Delanoë and Delanoë does not have priority in naming *Pneumocystis* from humans because the organism studied by the Delanoës was from the rat. The rat-derived *Pneumocystis* organism continues to be known as *P. carinii*, in keeping with principal III. As an additional historical note, Dr. Frenkel was the first investigator to understand the clear differences between human and rat-derived *Pneumocystis*, which were described in a landmark publication in 1976 (*3*). He proposed a change in nomenclature in which the name *Pneumocystis jiroveci* n. sp. applied to the human organisms and *Pneumocystis carinii* was retained for the rat organism. However, Frenkel did not attempt to follow ICBN procedures because at the time *Pneumocystis* was thought to be a protozoan. Nevertheless, this early paper established the idea of naming human *Pneumocystis*, *P. jiroveci*.

Dr. Hughes stated a concern over the possibility that the name change may cause “confusion and undue anxiety among the many thousands of HIV-infected patients who attend clinics.” Such concern is understandable. However, patients will have guidance in understanding the significance of the name change. Health-care providers will allay any fears that might be elicited by the application of the new name. The level of anxiety experienced by persons at risk of acquiring *Pneumocystis carinii* pneumonia (PCP) is more likely to decline than to increase. People may be relieved to learn that they are not going to catch PCP from a pet, for example.

Dr. Hughes suggested that the name *P. jiroveci* is unofficial because it has not yet been sanctioned by a body of experts that scrutinizes proposed name changes and has the power to either accept or reject them. This situation is not the case. The process by which new names are validated does not directly involve a body of experts. The International Botanical Congress does not evaluate names. Instead, the congress has established ICBN, which sets forth the procedures authors must follow to publish a valid new name. The scientific basis for the new name is included in the publication. In the case *of P. jiroveci*, abundant evidence shows that *P. carinii* and *P. jíroveci* are different species. This evidence, which also indicates that the genus *Pneumocystis* contains many additional species, has been reviewed extensively (*4*,*5*). Dr. Hughes gave the impression that that this evidence is exclusively molecular genetic data. In fact, the molecular genetic evidence is mirrored by clear biologic differences, the most dramatic being host species specificity.

As our knowledge of the biology and genetics of disease-causing microorganisms grows, openness to changes in the taxonomy and classification is needed. Given the impact such changes can have outside of the realm of basic science, the decision to accept the proposed changes in the nomenclature used for *Pneumocystis* has not been made frivolously. This decision is the result of a long and deliberate process that began almost 10 years ago, when data demonstrating that different mammalian hosts harbor different *Pneumocystis* species first began to appear. In 1994 and 2001, nomenclature issues were discussed at international meetings of the *Pneumocystis* community, with both physicians and research investigators present. In 1994, the data supporting new species were relatively limited. Consequently, a provisional tripartite nomenclature was adopted in lieu of recognizing new species (*6*). By 2001, however, the existence of multiple species and the necessity of assigning new species names were accepted by consensus. Because Frenkel had already published the name *P. jiroveci*, the suitability and validity of this name were also discussed. The new name was approved by consensus (*4*). We recognize that the results of these meetings do not necessarily reflect all opinions on the matter of *Pneumocystis* nomenclature, but we know of no better way to assess the majority opinion.

In endorsing the name *Pneumocystis jiroveci*, we hope to foster scientific understanding and communication. The tripartite name formerly used to denote the distinctness of this organism is not only cumbersome, it is inadequate because its meaning is not apparent and must be defined every time it is used. The arcane nature of the tripartite name tended to deprive the broad audience of persons interested in PCP of vital information, namely, a unique species of *Pneumocyctis* infects humans. By contrast*,* the new species name clearly states the uniqueness of *P. jiroveci*; a distinction is needed when assessing the significance of findings obtained by studies on other members of the genus. Recognition of this uniqueness will undoubtedly stimulate more research on this species. Communication will best be served by uniformity in nomenclature.

Frenkel has assigned a valid name to the *Pneumocystis* species found in humans. Ignoring this name on the grounds of inconvenience is not only unjustified, it is impractical. If names published in accordance with ICBN are not accepted, the field will have no recognized mechanism for conferring names, fostering the use of idiosyncratic, inadequate, and misleading names. Communication and progress will suffer as a result. Furthermore, if we choose to ignore critical changes in taxonomy for subjective reasons, when these changes are mandated by new and indisputable information, then taxonomy itself becomes meaningless and as a consequence, practitioners of public health, clinical medicine, and biomedical research might as well call any disease agent by whatever name they choose.

## References

[1] Stringer JR, Beard CB, Miller RF, Wakefield AE. A new name (*Pneumocystis jiroveci*) for *Pneumocystis* from humans. Emerg Infect Dis. 2002;8:891–6.1219476210.3201/eid0809.020096PMC2732539

[2] Frenkel JK. *Pneumocystis* pneumonia, an immunodeficiency-dependent disease (IDD): a critical historical overview. J Eukaryot Microbiol. 1999;46:89S–92.10519262

[3] Frenkel JK. *Pneumocystis jiroveci* n. sp. from man: morphology, physiology, and immunology in relation to pathology. Natl Cancer Inst Monogr. 1976;43:13–30.828240

[4] Stringer JR, Cushion MT, Wakefield AE. New nomenclature for the genus *Pneumocystis*. Supplement: Proceedings of the Seventh International Workshops on Opportunistic Protists, Cincinnati, Ohio, June 13–16, 2001. J Eukaryot Microbiol. 2001;Suppl:184s–9. 10.1111/j.1550-7408.2001.tb00512.x11906056

[5] Wakefield AE, Stringer JR, Tamburrini E, Dei-Cas E. Genetics, metabolism and host specificity of *Pneumocystis carinii.* Med Mycol. 1998;36:183–93.9988507

[6] Revised nomenclature for *Pneumocystis carinii*. The Pneumocystis Workshop. J Eukaryot Microbiol. 1994;41:121S–2.7804215
